# Unveiling species diversity within early-diverging fungi from China XV: Three new species of Cunninghamellaceae (Mucorales)

**DOI:** 10.3897/mycokeys.136.198771

**Published:** 2026-07-06

**Authors:** Wen-Xiu Liu, Heng Zhao, Qu-Hong Yan, Fei Li, Shu-Ting Geng, Hong-Yu Zou, Wei-Lai Lu, Pei-Jie Han, Shi Wang, Xiao-Yong Liu

**Affiliations:** 1 College of Life Sciences, Shandong Normal University, Jinan 250358, China Institute of Applied Ecology, Chinese Academy of Sciences Shenyang China; 2 CAS Key Laboratory of Forest Ecology and Silviculture, Institute of Applied Ecology, Chinese Academy of Sciences, Shenyang 110016, China College of Life Sciences, Shandong Normal University Jinan China; 3 National Engineering Research Center of Huangjiu, Zhejiang Guyuelongshan Shaoxing Wine Co., Ltd., Shaoxing, Zhejiang 312000, China Institute of Microbiology, Chinese Academy of Sciences Beijing China; 4 Zhejiang Traditional Shaoxing Huangjiu Science Research Institute, Zhejiang 312000, China National Engineering Research Center of Huangjiu, Zhejiang Guyuelongshan Shaoxing Wine Co., Ltd. Zhejiang China; 5 State Key Laboratory of Microbial Diversity and Innovative Utilization, Institute of Microbiology, Chinese Academy of Sciences, Beijing 100101, China Zhejiang Traditional Shaoxing Huangjiu Science Research Institute Zhejiang China

**Keywords:** Fungal diversity, molecular phylogeny, morphology, Mucorales, taxonomy

## Abstract

Fungi in the family Cunninghamellaceae are widely distributed, with soil being their primary habitat. During the isolation and identification of fungi from soil samples collected in Guangdong and Yunnan Provinces, China, three new species of the genera *Absidia* and *Cunninghamella* were discovered, namely *Absidia grisea***sp. nov**., *A. dendroidea***sp. nov**., and *Cunninghamella dimorpha***sp. nov**. Molecular phylogenetic analyses were performed using five loci: the internal transcribed spacer (ITS), large subunit (LSU), small subunit of ribosomal RNA gene (SSU rDNA), actin gene (*ACT*), and translation elongation factor 1-alpha gene (*TEF1α*). *Absidia grisea* is closely related to *A. purpurea*, and is distinguished by its gray colonies. *Absidia dendroidea***sp. nov**. is closely related to *A. stercoraria*, and is characterized by up to seven dendroidal sporangiophores from a single point on stolons. In contrast, *A. stercoraria* produces a maximum of five sporangiophores arising from a single point on its stolons, a number distinctly lower than that of *Absidia. dendroidea***sp. nov**. *Cunninghamella dimorpha* forms a sister clade with *C. blakesleeana*, and differs mainly by producing two distinct spore morphologies. To date, with the addition of these three new species described in this paper, the total number of accepted species in the genera *Absidia* and *Cunninghamella* has increased to 82 and 48, respectively.

## Introduction

The family Cunninghamellaceae belongs to Mucorales, Mucoromycetes, Mucoromycota. It was originally proposed by Naumov with the type genus *Chaetocladium* (Naumov 1935), and validly published by Benjamin (Benjamin 1959). When first established, the family comprised three genera *Cunninghamella*, *Sigmoideomyces*, and *Thamnocephalis* (Naumov 1935; Benjamin 1959). Later, many taxonomists assigned *Benjaminia*, *Chaetocladium*, *Mycotypha*, *Chaetocladium*, and *Phascolomyces* to Cunninghamellaceae (Hesseltine 1955a, 1955b; Benjamin 1959; Pidoplichko and Mil’ko 1971; Mil’ko 1974; Benny and Benjamin 1976). However, Cannon and Kirk (Fletcher 2008) included only *Cunninghamella* in this family. Currently, according to the Encyclopedia of Life (EOL) and the study by Zhao (Zhao et al. 2023), the family includes six genera, namely *Absidia*, *Chlamydoabsidia*, *Cunninghamella*, *Gongronella*, *Halteromyces*, and *Hesseltinella* (http://www.eol.org/, accessed on 16 April, 2026). Members of Cunninghamellaceae are widely distributed, among which *Absidia* and *Cunninghamella* are relatively common.

As a group of saprophytic fungi, *Absidia* species are commonly isolated from soil, and are also distributed in herbivore dung, and decaying organic matter (Zhang et al. 2018; Cordeiro et al. 2020; Lima et al. 2020; Vedprakash et al. 2021; Zhao et al. 2021b, 2023). The optimal growth temperature of this genus ranges from 20 to 42 °C (Hoffmann et al. 2007; Walther et al. 2013), and it is mainly distributed in warm and humid regions such as Brazil, China and Thailand (Crous et al. 2018, 2020; Cordeiro et al. 2020; Lima et al. 2020; Leitão et al. 2021; Lima et al. 2021; Zhao et al. 2021b; Zong et al. 2021). In China, the members of *Absidia* are mostly found in warm-climate regions such as Yunnan and Hainan, and are sparsely distributed in low-temperature areas, including Northeast and Northwest China (Zhao et al. 2022). *Absidia* can produce various secondary metabolites such as α-galactosidase, steroids and fatty acids (Kitahata et al. 1989; Davoust and Persson 1992; Kristanti et al. 2016; Kaczmarek et al. 2019; Chen et al. 2020; Zhao et al. 2021b), showing significant value in industrial and medicinal fields. Some species are also involved in the biotransformation of natural products, including flavones, flavanones and cresol red (Kristanti et al. 2016; Sordon et al. 2019). To date, 158 names of *Absidia* have been recorded in Index Fungorum (http://www.indexfungorum.org/, accessed on 16 April 2026), of which 135 are legitimate, and only 80 are accepted in Species Fungorum. (https://www.speciesfungorum.org/names/Names.asp, accessed on 16 April 2026).

Most species of the genus *Cunninghamella* are saprotrophic, with a small number being endophytic, and they are distributed in various habitats such as soil, air, and feces (Reed et al. 1988; Zhao et al. 2021a, 2023; Jiang et al. 2025). The optimal growth temperature for this genus is 23–28 °C (Zhao et al. 2021b), and it has a global distribution covering Antarctica, Brazil, India, Switzerland, Thailand, and other regions (Zheng and Chen 2001; Yu et al. 2015; Alves et al. 2017; Bragulat et al. 2017; Zhang et al. 2020; Suwannarach et al. 2021; Zhao et al. 2021b; Wang et al. 2022; Zhao et al. 2023). Strains of this genus can produce diverse metabolites, including fatty acids, terpenes, and nickel-iron carriers (Fakas et al. 2008; Alakhras et al. 2015; Zhao et al. 2021a; El-Seadawy et al. 2024), which are of significant value in biomedicine. To date, 75 names of *Cunninghamella* have been recorded in Index Fungorum (http://www.indexfungorum.org/, accessed on 16 April 2026), of which 67 are legitimate, and 47 are accepted in Species Fungorum (https://www.speciesfungorum.org/names/Names.asp, accessed on 16 April 2026).

Based on molecular phylogenetic and morphological evidence, this paper provides a systematic description of three new species of *Absidia* and *Cunninghamella* isolated from soil samples in Guangdong and Yunnan provinces. This paper constitutes the 15^th^ report in a serial study focusing on the diversity of early-diverging fungi in China (Jiang et al. 2025; Liu et al. 2026a, 2026b).

## Materials and methods

### Isolation and morphology

Soil samples were collected in Guangdong Province and Yunnan provinces in 2026. Fungal strains were isolated from the samples using a combination of the soil sprinkling method and the single spore method (Li et al. 2020; Zou et al. 2022). A soybean-sized portion of soil was evenly sprinkled onto Rose Bengal Chloramphenicol (RBC) agar plates (Corry et al. 1995) (RBC: peptone 5.00 g/L, glucose 10.00 g/L, KH_2_PO_4_ 1.00 g/L, MgSO_4_·7H_2_O 0.50 g/L, rose red 0.05 g/L, chloramphenicol 0.10 g/L, agar 15.00 g/L) and incubated at 25 °C in darkness for 2–5 days. Under a stereomicroscope (Olympus SZX10, Tokyo, Japan), spores of the target strains were picked using a sterile inoculation needle and transferred to fresh Potato Dextrose Agar (PDA) medium (PDA: glucose 20 g/L, potato 200 g/L, agar 20 g/L). Colony morphology (obverse and reverse) was photographed using a digital camera (Canon PowerShot G7X, Canon, Tokyo, Japan). All strains were preserved in 10% sterile glycerol at 4 °C. Microscopic morphology of the strains was observed using an optical microscope (Olympus BX53 Manufacturer: Olympus Corporation Tokyo, Japan) and captured with a high-definition digital camera (Olympus DP80 Manufacturer: Olympus Corporation, Tokyo, Japan). Morphological traits were measured using Digimizer software (v.5.6.0); each indicator was measured at least 30 times. The living cultures were preserved at the China General Microbiological Culture Collection Center, Beijing, China (**CGMCC**). Equivalent strains were stored at Shandong Normal University (**XG**). Dry cultures of types were kept at the Herbarium Mycologicum Academiae Sinicae, Beijing, China (**Fungarium**; **HMAS**). The taxonomic information was deposited at the Fungal Names repository (https://nmdc.cn/fungalnames, accessed on 15 March 2025).

### DNA extraction, PCR amplification, and sequencing

Total genomic DNA of the strains was extracted using the modified cetyltrimethylammonium bromide (CTAB) method described (Doyle et al. 1990; Guo et al. 2008). After the strains fully covered the plate, 0.3 g of mycelium was scraped with a scalpel under aseptic conditions and transferred into a sterile 1.5 mL centrifuge tube. Pre-warmed CTAB buffer at 65 °C was added, and the mixture was ground thoroughly with steel beads in a grinder. The centrifuge tube was incubated in a 65 °C water bath for 2 h, with inversion and mixing every 30 min. Centrifugation was performed at 12,000 rpm for 10 min at 4 °C. The supernatant was transferred to a new centrifuge tube, mixed with an equal volume of chloroform-isoamyl alcohol mixture (24:1, v/v), rotated and homogenized on a shaker for 20 min, followed by a second centrifugation. The supernatant was transferred to a new centrifuge tube, and pre-chilled isopropanol at 4 °C was added. After standing for 20 min, a third centrifugation was conducted. The pellet was washed with anhydrous ethanol three times and dissolved in 50 μL of ddH_2_O.

DNA was extracted using an extraction kit (Changchun GeneOn BioTech Co., Ltd. Changchun, China). The ITS, LSU, *TEF1α*, *ACT* and SSU regions were amplified using the primers and programs listed in Table 1. The PCR mixture (25 μL final volume) was prepared using 12.5 μL of 2× Hieff Canace Plus PCR Master Mix with dye (Yeasen Biotechnology, Cat No. 10154ES03. Shanghai, China), to which 9.5 μL of ddH2O, 1 μL of each primer (10 μM), and 1 μL of genomic DNA template (1 ng/μL) were added. PCR products were subjected to 1% agarose gel electrophoresis and observed under UV light (Shenhua Science Technology Co., Ltd. Hangzhou, China) at 254 nm (Zhang et al. 2022).

**Table 1.** PCR primers and amplification programs were employed in this study.

Loci	PCR Primers	Primer Sequence (5'–3')	PCR Cycles	References
ITS	ITS5	GGA AGT AAA AGT CGT AAC AAG G	95 °C 5 min; (95 °C 30 s, 55 °C 30 s, 72 °C 1 min) × 35 cycles; 72 °C 10 min	White et al. (1990)
	ITS4	TCC TCC GCT TAT TGA TAT GC		
LSU	LR0R	GTA CCC GCT GAA CTT AAG C	95 °C 5 min; (95 °C 50 s, 47 °C 30 s, 72 °C 1.5 min) × 35 cycles; 72 °C 10 min	Hurdeal et al. (2023)
	LR5	TCC TGA GGG AAA CTT CG		
*TEF1α*	EF1-983F	GCYCCYGGHCAYCGTGAYTTYAT	95 °C 5 min; (95 °C: 30 s, 55 °C: 60 s, 72 °C: 60 s) × 30 cycles; 72 °C 10 min	Jaklitsch et al. (2005); Rehner and Buckley (2005)
	TEF1LLErev	AAC TTG CAG GCA ATG TGG		
*ACT*	ACT-1	TGG GAC GAT ATG GAI AAI ATC TGG CA	95 °C 3 min; (95 °C: 60 s, 55 °C: 60 s, 72 °C: 1 min) × 30 cycles; 72 °C 10 min	Voigt and Wöstemeyer (2000)
	ACT-4R	TC ITC GTA TIC TIG CTI IGA IAT CCA CA T		
SSU	NS1	GTA GTC ATA TGC TTG TCT CC	95 °C 5 min; (94 °C: 60 s, 54 °C: 50 s, 72 °C: 1 min) × 37 cycles; 72 °C 10 min	White et al. (1990)
	NS4	CTT CCG TCA ATT CCT TTA AG		

Upon obtaining the sequencing results, sequence analysis, alignment and assembly were performed using MEGA 7.0 software. The processed sequences were subjected to BLAST searches against the database of the National Center for Biotechnology Information (NCBI, USA, http://blast.ncbi.nlm.nih.gov/). Sequences of the three taxa obtained in this study after alignment have been uploaded and deposited in the GenBank database of NCBI, USA.

### Phylogenetic analyses

Sequences were obtained from the GenBank database of the National Center for Biotechnology Information (NCBI) (see Suppl. materials 1, 2 for details, accessed on 21 March 2026). A genus was selected as the outgroup, and Geneious Prime v. 2025.0.2 software (https://www.geneious.com/features/prime/, accessed on 21 May 2025) was used to perform sequence alignment on the nucleic acid sequences of the new taxa of the genus and the reference sequences retrieved from public databases. To reconstruct the phylogenetic relationships, in this study, single-gene markers were analyzed separately. For the genus *Absidia*, the combined matrix of ITS-LSU-SSU-*ACT*-*TEF1α* was used, while for the genus *Cunninghamella*, the combined matrix of ITS-LSU-*TEF1α* was employed, using the Maximum likelihood (ML) and Bayesian inference (BI) methods. Maximum likelihood (ML) analysis was performed using RAxML-NG v1.1.2 with default parameters. Bayesian inference (BI) analysis was carried out in MrBayes v3.2.7a, employing the fast bootstrap algorithm and automatic stop criterion. The burn-in fraction was set to 0.25, and posterior probabilities (PP) were calculated from the remaining trees. The results of all phylogenetic trees were visualized using iTOL (https://itol.embl.de/; accessed 24 March 2026), and the final layout adjustments were completed with Adobe Illustrator CC 2019.

## Results

### Phylogeny

Phylogenetic analysis of the genus *Absidia* was based on 114 strains, covering 68 species of *Absidia*, with *Cunninghamella blakesleeana* (CBS 133.27 and CBS 782.68) and *C. elegans* (CBS 160.28 and CBS 167.53) selected as the outgroup. The dataset comprised a total of 5221 concatenated sequence characters, covering five gene regions: ITS (1–1229), LSU (1230–2314), SSU (2315–3412), *ACT* (3413–4367), and *TEF1α* (4368–5221). Of these characters, 2909 were constant, 536 were variable but parsimony-uninformative, and 1776 were parsimony-informative.

Phylogenetic analysis of the genus *Cunninghamella* was based on 91 strains, covering 48 species of *Cunninghamella*, with *Backusella oblongispora* (CBS 569.70) selected as the outgroup. The dataset comprised a total of 4245 concatenated sequence characters, covering three gene regions: ITS (1–1465), LSU (1466–2855), and *TEF1α* (2856–4245). Of these characters, 1836 were constant, 539 were variable but parsimony-uninformative, and 1870 were parsimony-informative.

**Figure 1.**
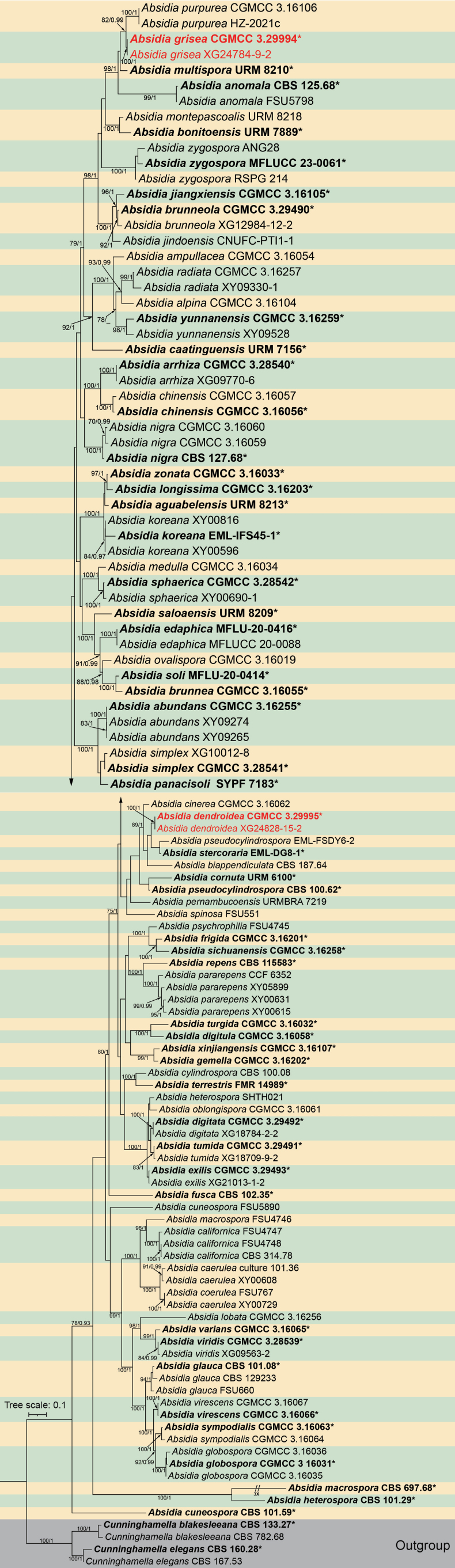
The maximum likelihood (ML) phylogenetic tree of the genus *Absidia* was constructed using concatenated sequences of ITS, LSU, SSU, ACT, and TEF-1α, with *Cunninghamella blakesleeana* (CBS 133.27 and CBS 782.68) and *C. elegans* (CBS 160.28 and CBS 167.53) as the outgroup. Node support values are indicated as ML bootstrap values (≥70%) / Bayesian posterior probabilities (≥0.90). Ex-type / ex-holotype strains are highlighted in bold black font with an asterisk (*); the four novel species strains isolated in this study are highlighted in red. A double slash “//” denotes shortened branches, with “×” indicating the fold of shortening. The phylogenetic tree is divided into two parts connected by arrows at the top and bottom. The scale bar represents the number of nucleotide substitutions per site.

**Figure 2.**
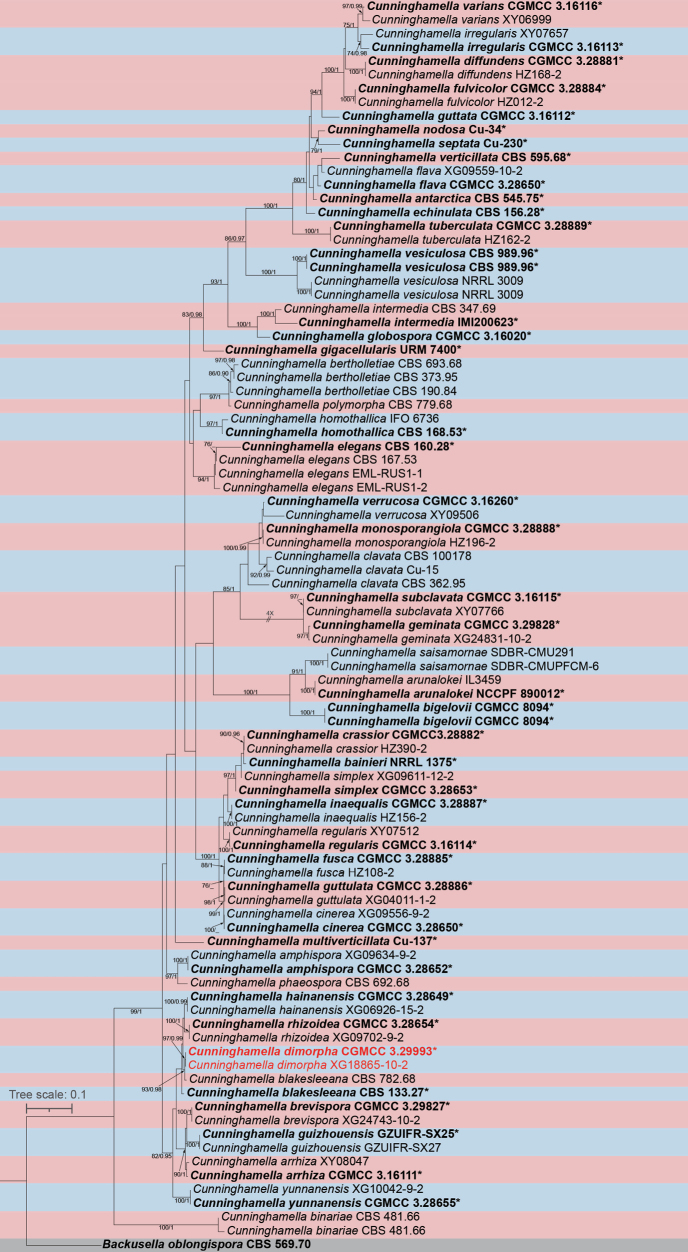
The maximum likelihood (ML) phylogenetic tree of the genus *Cunninghamella* was constructed using concatenated sequences of ITS, LSU and TEF-1α, with *Backusella oblongispora* (CBS 569.70) as the outgroup. Node support values are indicated as ML bootstrap values (≥70%) / Bayesian posterior probabilities (≥0.90). Ex-type / ex-holotype strains are highlighted in bold black font with an asterisk (*); the two novel species strains isolated in this study are highlighted in red. A double slash “//” denotes shortened branches, with “×” indicating the fold of shortening. The scale bar represents the number of nucleotide substitutions per site.

The topologies of the maximum likelihood (ML) trees were congruent with those of the Bayesian inference (BI) trees for both genera; thus, the ML trees were finally selected for comprehensive visualization (Figs 1, 2). Four *Absidia* strains and two *Cunninghamella* strains obtained in this study formed three highly supported, distinct clades in the phylogenetic trees, each receiving full statistical support.

### Taxonomy

#### Absidia grisea

Taxon classificationFungiMucoralesCunninghamellaceae

W.X. Liu, H. Zhao & X.Y. Liu sp. nov.

68CE0E2F-0A5D-540E-8B1D-254F728DF9FE

Fungal Names: FN 573744

Fig. 3

##### Type.

China • Guangdong Province, Maoming City, Xinyi City, Provincial Highway 360 (22.374354°N, 111.107367°E, altitude 366.2 m), from soil, 29 December 2025, W. X. Liu, holotype HMAS 354477, ex-holotype living culture CGMCC 3.29994 (=XG24784-9-1).

##### Etymology.

The epithet *grisea* (Lat.) refers to gray colonies.

##### Description.

Colonies on PDA at 25 °C for 6 days, reaching 60 mm in diameter. Hyphae initially white, becoming gray with age, aseptate when young, septate at maturity, 2.6–11.4 μm wide. Stolons septate, branched, hyaline or dark brown, 1.7–8.7 μm wide. Rhizoids well-developed, root-like. Sporangiophores arising from stolons, erect or curved, unbranched, monopodially branched or sympodially branched, single or 2–5 in whorls, 36.1–321.3 μm long, 1.4–5.8 μm wide, with a septum 10.2–16.2 μm below the apophysis. Sporangia pyriform, hyaline or brown, smooth-walled, deliquescent, 14.3–38 μm long, 11–28 μm wide. Columellae globose, hyaline, smooth-walled, 5.9–20.3 μm long, 5.1–21.5 μm wide. Projections present or absent; if present, conspicuous and pointed. Apophyses distinct, funnel-shaped, 1.7–10.8 μm wide at the base, and 8.9–26.5 μm wide at the top. Sporangiospores globose or ovoid, hyaline, smooth-walled, 2.6–4.9 μm long, 2.0–4.3 μm wide.

**Figure 3.**
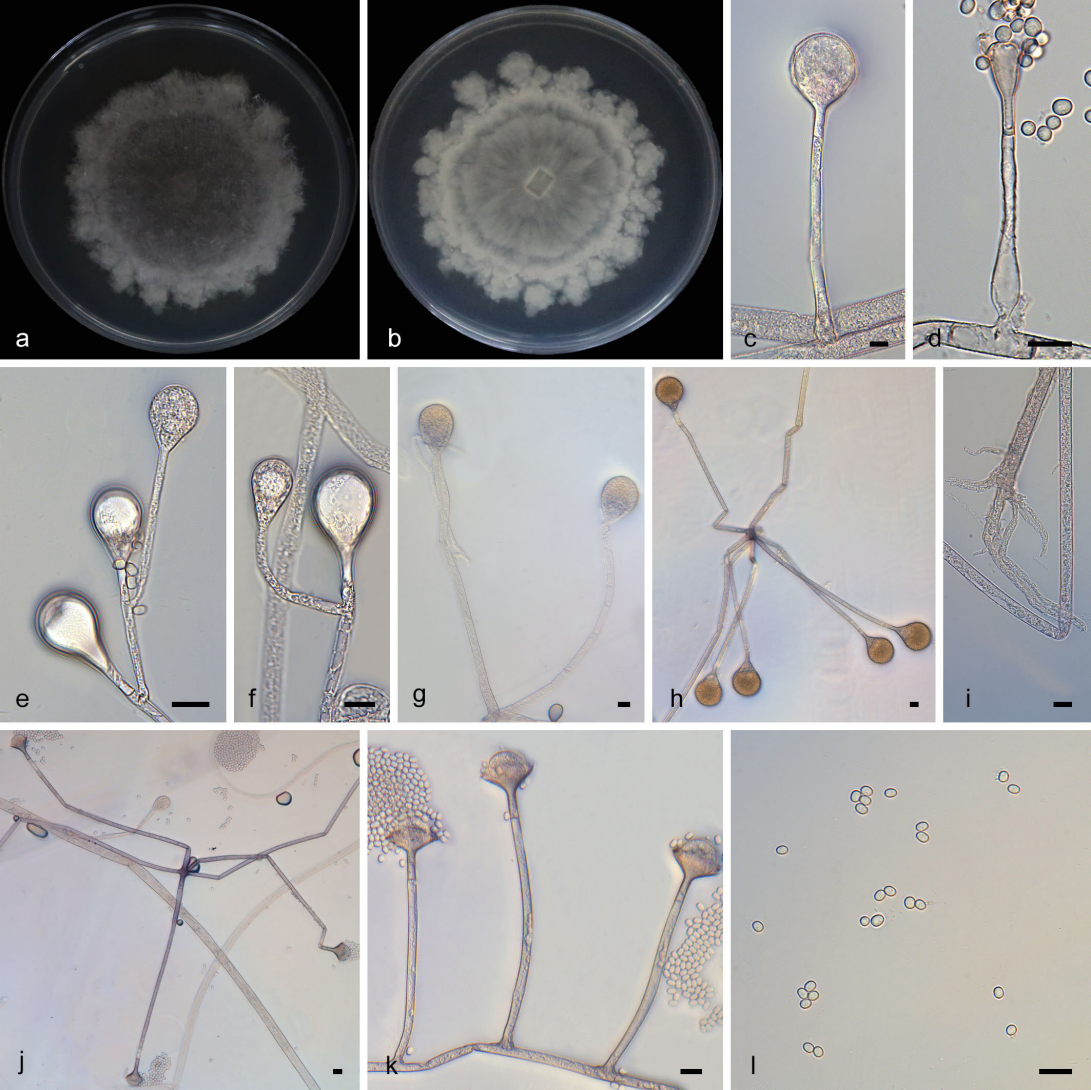
Morphologies of *Absidia grisea* (ex-holotype CGMCC 3.29994). **a, b**. Colonies on PDA (a. obverse; b. reverse); **c, e–h**. Sporangia; **d, j, k**. Columellae; **i**. Rhizoids; **l**. Sporangiospores. Scale bars: 10 μm (**c–l**).

##### Additional strains examined.

China • Guangdong Province, Maoming City, Xinyi City, Provincial Highway 360 (22.374354°N, 111.107367°E, altitude 366.2 m), from soil, 29 December 2025, W. X. Liu, living culture XG24784-9-2; ibid., from soil, 29 December 2025, W. X. Liu, living culture XG24784-11.

##### Notes.

According to the ITS–LSU–SSU–*Act*–*TEF1α* phylogenetic tree, strains CGMCC 3.29994 and XG24784-9-2 cluster together to form a single clade with a support value of MLBS/BIPP = 100/1.00 (Fig. 1). It is closely related to *Absidia purpurea* (Zhao et al. 2023). ITS similarity: 98.6% (9/577 number of nucleotide differences); LSU, SSU, *ACT*, and *TEF 1α* sequences: Not available. Morphologically, mature colonies of the two strains are gray, with gray hyphae and sympodial branching. In contrast, mature colonies of *Absidia purpurea* are pale quaker drab, with violet-blue hyphae and no sympodial branching. Based on molecular phylogenetic and morphological evidence, these two strains are described as a new species of Absidia in this study, named *A. grisea*.

#### Absidia dendroidea

Taxon classificationFungiMucoralesCunninghamellaceae

W.X. Liu, H. Zhao & X.Y. Liu sp. nov.

59F16315-374B-5394-BD42-000A958CDD87

Fungal Names: FN 573745

Fig. 4

##### Type.

China • Guangdong Province, Maoming City, Huazhou City, 380 Xiang Road. (22.096213°N, 110.444750°E, altitude 95.5 m), from soil, 29 December 2025, W. X. Liu, holotype HMAS 354478, ex-holotype living culture CGMCC 3.29995 (=XG24828-15-1).

##### Etymology.

The epithet *dendroidea* (Lat.) refers to dendroidally branched sporangiophores.

##### Description.

Colonies on PDA at 25 °C for 6 days, reaching 53 mm in diameter. Hyphae initially white, gradually becoming yellowish brown with age; aseptate when young, septate at maturity, 3.7–12.1 μm wide. Stolons septate, branched, hyaline, 3.1–8.8 μm wide. Rhizoids absent. Sporangiophores arising from stolons, erect or curved, unbranched or simply branched, sometimes with a swelling, solitary or 2–7 in whorls, 20.5–234.9 μm long, 2.3–6.2 μm wide, with a septum 6.6–14.5 μm below the apophysis. Sporangia globose, hyaline or brown, smooth-walled, deliquescent, 8.6–34.3 μm long, 8.6–35.3 μm wide. Columellae globose, hyaline, smooth-walled, 3.1–22.2 μm long, 3.4–18.8 μm wide. Projections present or absent; if present, conspicuous and pointed. Apophyses distinct, funnel-shaped, 4.0–12.4 μm high, 2.5–6.4 μm wide at the base, and 7.8–22 μm wide at the top. Sporangiospores ellipsoid, hyaline, smooth-walled, 3.6–5.3 μm long, 1.6–2.9 μm wide.

**Figure 4.**
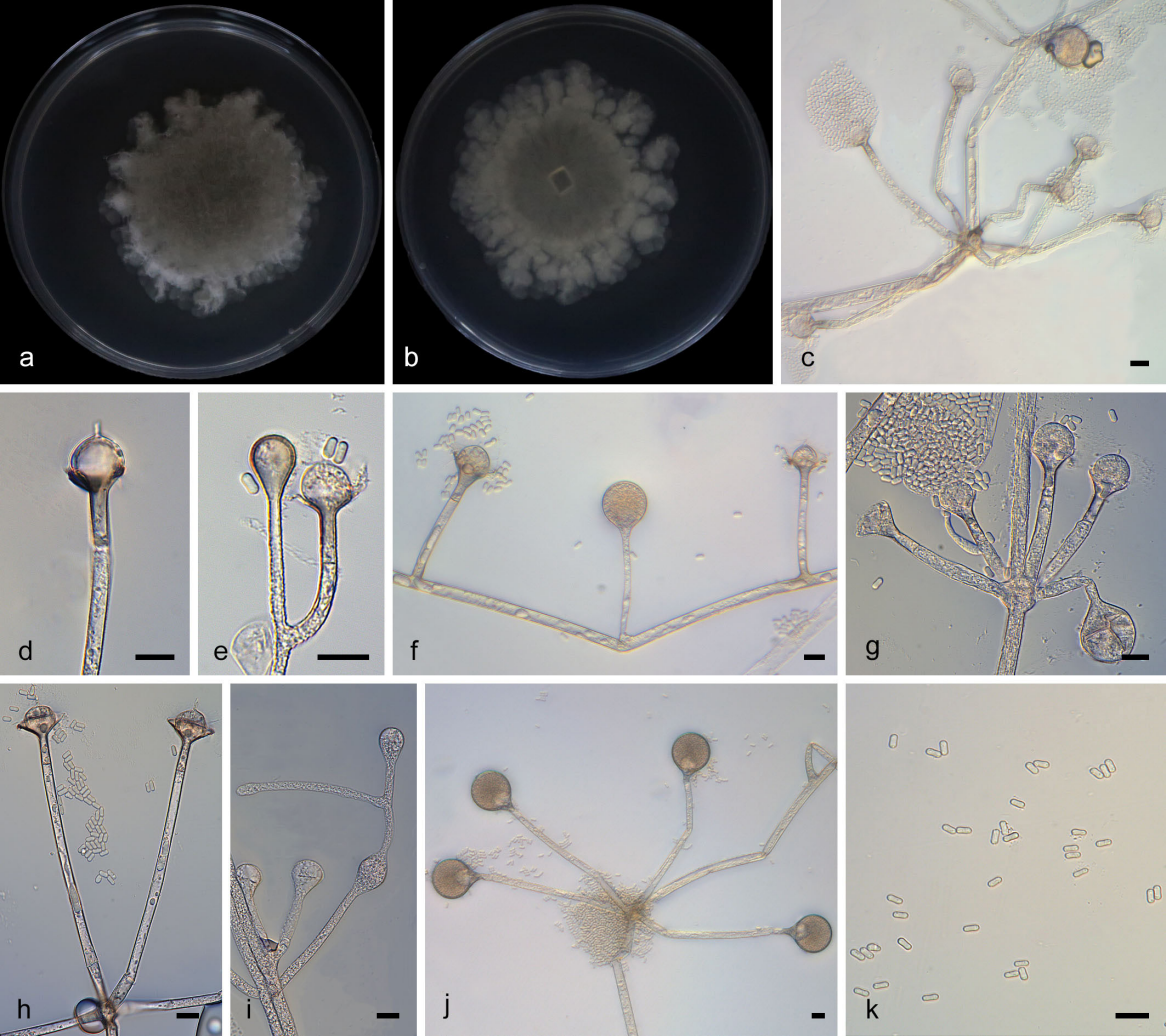
Morphologies of *Absidia dendroidea* (ex-holotype CGMCC 3.29995). **a, b**. Colonies on PDA (**a**. Obverse; **b**. Reverse); **c–e, g–i**. Columellae; **f, j**. Sporangia; **k**. Sporangiospores. Scale bars: 10 μm (**c–k**).

##### Additional strains examined.

China • Guangdong Province, Maoming City, Huazhou City, 380 Xiang Road (22.096213°N, 110.444750°E, altitude 95.5 m), from soil, 29 December 2025, W. X. Liu, living culture XG24828-15-2; China • Guangdong Province, Yunfu City, Luoding City, Provincial Highway 479 (22.622250°N, 111.432883°E, altitude 226.4 m), from soil, 24 December 2025, W. X. Liu, living culture XG24767-2.

##### Notes.

According to the ITS–LSU–SSU–*ACT*–*TEF1α* phylogenetic tree, strains CGMCC 3.29995 and XG24828-15-2 cluster together to form a single clade with a support value of MLBS/BIPP = 100/1.00 (Fig. 1), It is closely related to *Absidia stercoraria* (Li et al. 2016) and *A. pseudocylindrospora* (Hesseltine and Ellis 1961). *A. stercoraria*: ITS similarity: 98.6% (9/577 number of nucleotide differences); LSU similarity: 97.0% (19/626 number of nucleotide differences); SSU similarity: 97.0% (23/1063 number of nucleotide differences); *ACT* similarity: 100.0% (0/619 number of nucleotide differences) and *TEF 1α* similarity: 99.0% (6/448 number of nucleotide differences); *A. pseudocylindrospora*: ITS similarity: 96.0% (19/492 number of nucleotide differences); LSU similarity: 98.0% (10/611 number of nucleotide differences); SSU similarity: 97.0% (30/944 number of nucleotide differences); *ACT* similarity: 90.0% (62/603 number of nucleotide differences) and *TEF 1α* sequences: Not available. Morphologically, the two strains produce numerous structures in whorls at the same position on the stolons, with up to 7 in number, whereas those of *Absidia stercoraria* are up to 5. Based on molecular phylogenetic and morphological evidence, the two strains are described as a new species of the genus *Absidia* in this study, and named *A. dendroidea*.

#### Cunninghamella dimorpha

Taxon classificationFungiMucoralesCunninghamellaceae

W.X. Liu, H. Zhao & X.Y. Liu sp. nov.

206FA2F8-98CC-5D40-8307-C937E110F067

Fungal Names: FN 573746

Fig. 5

##### Type.

China • Yunnan Province, Xishuangbanna Dai Autonomous Prefecture, Jinghong City, Gasa Street, G219 (Xijing Line). (21.933295°N, 100.689181°E, altitude 680.5 m), from soil, 8 June 2025, W. X. Liu, holotype HMAS 354480, ex-holotype living culture CGMCC 3.29993 (=XG18865-10-1).

##### Etymology.

The epithet *dimorpha*

**Figure 5.**
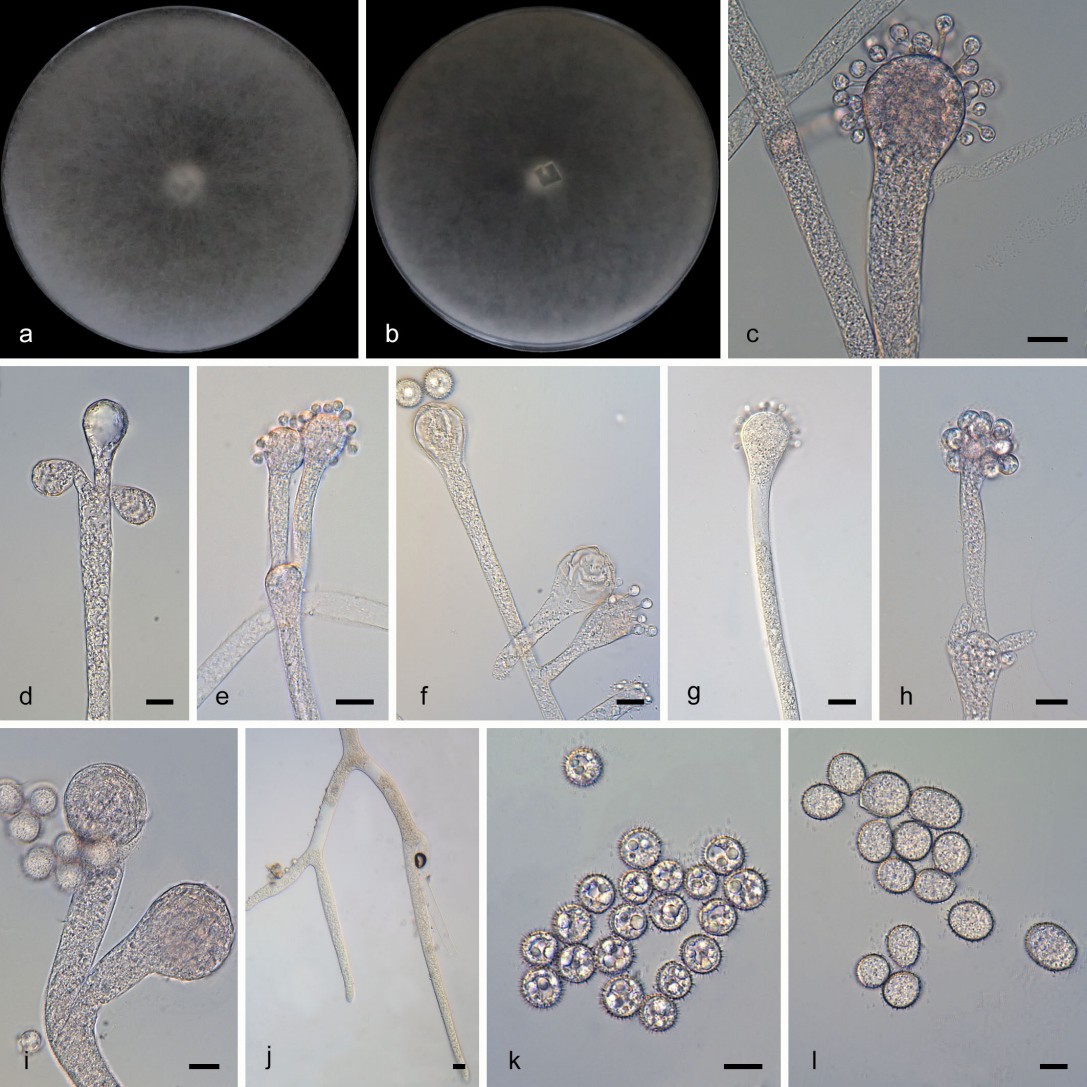
Morphologies of *Cunninghamella dimorpha* (ex-holotype CGMCC 3.29993). **a, b**. Colonies on PDA (**a**. Obverse; **b**. Reverse); **d–f, h, i**. Sporangiophores with branching patterns; **c, g**. Vesicles with sporangiola; **j**. Rhizoids; **k, l**. Sporangiola. Scale bars: 10 μm (**c–l**).

(Lat.) refers to two types of sporangia.

##### Description.

Colonies initially white, gradually turning dark gray with increasing incubation time, floccose. Hyphae aseptate when young, septate at maturity, branched, 5.7–17.0 μm wide. Rhizoids present. Sporangiophores arising from stolons, erect or slightly curved, unbranched or with simple branches, simple verticils present, gradually widening from the base upwards, aseptate, 5.0–13.5 μm wide. Vesicles subglobose to globose, hyaline, rough-walled, 12.4–31.4 μm long, 13.2–28.4 μm wide. Sporangiola borne on vesicles, globose, ellipsoid or ovoid, spinose, brown, 5.8–19.7 μm long, 5.6–15.3 μm wide.

##### Additional strains examined.

China • Yunnan Province, Xishuangbanna Dai Autonomous Prefecture, Jinghong City, Gasa Street, G219 (Xijing Line) (21.933295°N, 100.689181°E, altitude 680.5 m), from soil, 8 June 2025, W. X. Liu, living culture XG18865-10-2; China, Yunnan Province, Xishuangbanna Dai Autonomous Prefecture, Mengla County, Mengman Town, Mengman Service Area (21.318024°N, 101.298125°E, altitude 578.0 m), from soil, 20 May 2025, W. X. Liu, living culture XG18825-10; ibid., from soil, 20 May 2025, W. X. Liu, living culture XG18825-13.

##### Notes.

According to the ITS–LSU–SSU–*Act*–*TEF1α* phylogenetic tree, strains CGMCC 3.29993 and XG18865-10-2 cluster together to form a single clade with a support value of MLBS/BIPP = 97/0.99 (Fig. 2). ITS similarity: 98.0% (16/717 number of nucleotide differences); LSU similarity: 99.0% (6/1034 number of nucleotide differences) and *TEF 1α* similarity: 99.0% (4/314 number of nucleotide differences). It is sister to *Cunninghamella blakesleeana* (Liu et al. 2005). Morphologically, two types of spores were observed in the two strains, whereas *C. blakesleeana* possesses only one spore type. Based on molecular phylogenetic and morphological evidence, this study describes the two strains as a new species of the genus *Cunninghamella*, named *C. dimorpha*.

## Discussion

The discovery of new species in the family Cunninghamellaceae further confirms that this family exhibits high species diversity, especially within the diverse ecological environments of China. Phylogenetic analyses indicate that the three new species described in this study each form independent, well-supported clades (Figs 1, 2).

*Absidia grisea* is closely related to *Absidia purpurea*. In comparison, colonies and hyphae of *A. grisea* are gray and show sympodial branching, whereas colonies of *A. purpurea* are pale grayish brown, with violet-blue hyphae and no sympodial branching. *Absidia dendroidea* is phylogenetically close to *Absidia stercoraria*. Morphologically, *A. dendroidea* forms dendroid whorls, with up to seven structures produced in whorls at the same position on stolons, while *A. stercoraria* produces a maximum of five such structures. *Cunninghamella dimorpha* is sister to *Cunninghamella blakesleeana*. The most significant difference between them is that the former produces two types of spores, whereas the latter produces only one spore type.

Most species of the family Cunninghamellaceae are saprotrophic fungi that play crucial roles in terrestrial ecosystems. They are involved in the decomposition of plant residues, animal dung, and other organic materials, thereby promoting nutrient cycling and maintaining soil fertility (Matruchot 1903; Reed et al. 1988; Richardson 2009; Vedprakash et al. 2021). Some members of Cunninghamellaceae are capable of producing chitosan and organic acids (Kitahata et al. 1989; Davoust and Persson 1992; Fakas et al. 2008; Alakhras et al. 2015; Kaczmarek et al. 2019; Chen et al. 2020), while certain species can synthesize polyunsaturated fatty acids, bioactive alkaloids, and other compounds, endowing them with significant research value in industrial and biomedical applications (Kristanti et al. 2016). In addition, some species in this family are opportunistic human pathogens that affect immunocompromised individuals and can cause mucormycosis (Reed et al. 1988; Gomes et al. 2011; Kwon-Chung 2012; Yu et al. 2015). As the primary habitat of Cunninghamellaceae, soil provides a stable environment rich in organic matter for these fungi (Richardson 2009). In the present study, new species were isolated from soils in Guangdong and Yunnan provinces, suggesting that warm, humid environments with high organic matter content favor the survival of Cunninghamellaceae strains. This is consistent with previous findings that Cunninghamellaceae species are more diverse in tropical and subtropical regions (Zheng and Chen 2001; Liu et al. 2005; Alves et al. 2017; Bragulat et al. 2017; Nguyen et al. 2017; Cordeiro et al. 2020; Lima et al. 2020; Zhang et al. 2020; Leitão et al. 2021; Vedprakash et al. 2021; Wang et al. 2022; Zhao et al. 2023). In summary, this study enriches the species diversity of Cunninghamellaceae, clarifies the phylogenetic relationships of the new taxa, and lays a foundation for further taxonomic investigations of this family.

## Supplementary Material

XML Treatment for Absidia grisea

XML Treatment for Absidia dendroidea

XML Treatment for Cunninghamella dimorpha

## Additional information

### Artificial Intelligence (AI) use

The authors accept full responsibility for the content of the manuscript, including the disclosure of any use of AI.

No AI tools were used in the preparation of this manuscript.

### Author ORCIDs

W.-X. Liu https://orcid.org/0009-0002-3283-3704

H. Zhao https://orcid.org/0000-0003-2938-5613

F. Li https://orcid.org/0009-0002-7969-6671

S.-T. Geng https://orcid.org/0009-0005-4074-0657

H.-Y. Zou https://orcid.org/0009-0005-6469-4667

W.-L. Lu https://orcid.org/0000-0003-1657-1273

P.-J. Han https://orcid.org/0000-0001-8342-739X

S. Wang https://orcid.org/0000-0002-7376-7638

X.-Y. Liu https://orcid.org/0000-0002-8808-010X

### Data availability

The sequences of this study have been submitted to the NCBI database (https://www.ncbi.nlm.nih.gov/, 21 March 2026) with accession numbers shown in Suppl. materials 1, 2.

## Supplementary materials

Supplementary material 1 GenBank accession numbers of *Absidia* sequences used in this study Data type docx https://binary.pensoft.net/file/1708998This dataset is made available under the Open Database License (http://opendatacommons.org/licenses/odbl/1.0/). The Open Database License (ODbL) is a license agreement intended to allow users to freely share, modify, and use this Dataset while maintaining this same freedom for others, provided that the original source and author(s) are credited. Wen-Xiu Liu, Heng Zhao, Qu-Hong Yan, Fei-Li, Shu-Ting Geng, Hong-Yu Zou, Wei-Lai Lu, Pei-Jie Han, Shi Wang, Xiao-Yong Liu

Supplementary material 2 GenBank accession numbers of *Cunninghamella* sequences used in this study https://doi.org/10.3897/mycokeys.136.198771.suppl2 Data type docx https://binary.pensoft.net/file/1708999This dataset is made available under the Open Database License (http://opendatacommons.org/licenses/odbl/1.0/). The Open Database License (ODbL) is a license agreement intended to allow users to freely share, modify, and use this Dataset while maintaining this same freedom for others, provided that the original source and author(s) are credited. Wen-Xiu Liu, Heng Zhao, Qu-Hong Yan, Fei-Li, Shu-Ting Geng, Hong-Yu Zou, Wei-Lai Lu, Pei-Jie Han, Shi Wang, Xiao-Yong Liu
