# Supplementary material for: Unveiling species diversity within early-diverging fungi from China XV: Three new species of Cunninghamellaceae (Mucorales)
Source: MycoKeys. 2026 Jul 6;136:119–38. doi: 10.3897/mycokeys.136.198771 (PMC13366047; doi:10.3897/mycokeys.136.198771)
Supplement: Supplementary material 2 — GenBank accession numbers of Cunninghamella sequences used in this study [file mycokeys-136-119-s002.docx]

**Table S2**. GenBank accession numbers of *Cunninghamella* and *Backusella* strains in this study.

| **Species** | **Strains** | **GenBank Accession Numbers** | | |
| --- | --- | --- | --- | --- |
|  |  | **ITS** | **LSU** | ***TEF1α*** |
| *C. amphispora* | CGMCC 3.28652* | PV089203 | PV123104 | PV200769 |
|  | XG09634-9-2 | PV089204 | PV123105 | PV200770 |
| *C. antarctica* | CBS 545.75 ^T^ | JN205893 | JN206597 | KJ156492 |
| *C. arrhiza* | CGMCC 3.16111 ^T^ | OL678142 | PQ399916 | NA |
|  | XY08047 | OL678143 | NA | NA |
| *C. arunalokei* | IL3459 | MN431159 | MN431158 | NA |
|  | NCCPF 890012 ^T^ | NR_177485 | NG153887 | NA |
| *C. bainieri* | CBS 481.66 | MH858865 | MH870507 | KJ156495 |
|  | CGMCC 8094 ^T^ | KJ013403 | KJ013405 | KJ395944 |
|  | NRRL 1375 ^T^ | AF254935 | NA | NA |
| *C. bertholletiae* | CBS 190.84 | JN205878 | HM849701 | NA |
|  | CBS 373.95 | JN205873 | NA | KJ156497 |
|  | CBS 693.68 | AF254931 | MH870924 | KJ156490 |
| *C. bigelovii* | CGMCC 8094 ^T^ | KJ013403 | KJ013405 | KJ395944 |
| *C. binariae* | CBS 481.66 | MH858865 | MH870507 | KJ156495 |
| *C. blakesleeana* | CBS 133.27 ^T^ | NR119974 | MH866397 | KJ156479 |
|  | CBS 782.68 | JN205869 | MH870950 | KJ156478 |
| *C. brevispora* | CGMCC3.29827 ^T^ | PZ272631 | PZ278609 | NA |
|  | XG24743-10-2 | PZ272630 | PZ278610 | NA |
| *C. cinerea* | CGMCC 3.28650* | PV089197 | PV123098 | PV172612 |
|  | XG09556-9-2 | PV089198 | PV123099 | PV172613 |
| *C. clavata* | CBS 100178 | JN205890 | JN206604 | KJ156477 |
|  | Cu-15 | AF254942 | NA | NA |
|  | CBS 362.95 | JN205891 | NA | NA |
| *C. crassior* | CGMCC3.28882 ^T^ | PV239680 | PV235924 | PV254891 |
|  | HZ390-2 | PV239681 | PV235925 | PV254892 |
| *C. diffundens* | CGMCC3.28881 ^T^ | PV239676 | PV235920 | PV254889 |
|  | HZ168-2 | PV239677 | PV235921 | PV254890 |
| ***C. dimorpha*** | **CGMCC 3.29993** | **PZ272629** | **PZ278607** | **PZ291124** |
|  | **XG18865-10-2** | **PZ272630** | **PZ278608** | **PZ291125** |
| *C. echinulata* | CBS 156.28 ^T^ | JN205895 | JN939199.1 | KJ156500 |
| *C. elegans* | CBS 160.28 ^T^ | AF254928 | NR_154747 | KJ156470 |
|  | CBS 167.53 | JN205882 | HM849700 | KJ156494 |
|  | EML-RUS1-1 | MF806023 | MF806027 | NA |
|  | EML-RUS1-2 | MF806021 | MF806028 | NA |
| *C. flava* | CGMCC 3.28651 ^T^ | PV089199 | PV123100 | PV200765 |
|  | XG09559-10-2 | PV089200 | PV123101 | PV200766 |
| *C. fulvicolor* | CGMCC3.28884 ^T^ | PV239666 | PV235910 | PV254879 |
|  | HZ012-2 | PV239667 | PV235911 | PV254880 |
| *C. fusca* | CGMCC3.28885 ^T^ | PV239664 | PV235908 | PV254877 |
|  | HZ108-2 | PV239665 | PV235909 | PV254878 |
| *C. geminata* | CGMCC3.29828 ^T^ | PZ272633 | PZ278611 | PZ291126 |
|  | XG24831-10-2 | PZ272634 | PZ278612 | PZ291127 |
| *C. globospora* | CGMCC 3.16020 ^T^ | MW264073 | MW264132 | NA |
| *C. gigacellularis* | URM 7400 ^T^ | NR_168760 | NG_068773 | NA |
| *C. guizhouensis* | GZUIFR-SX25 ^T^ | MN908596 | MN908599 | MN912633 |
|  | GZUIFR-SX27 | MN908598 | MN908601 | MN912635 |
| *C. guttata* | CGMCC 3.16112 ^T^ | OL678144 | PQ399917 | NA |
| *C. guttulata* | CGMCC3.28886 ^T^ | PV235930 | PV239686 | PV254893 |
|  | XG04011-1-2 | PV235931 | PV239687 | PV254894 |
| *C. hainanensis* | CGMCC 3.28649* | PV089195 | PV123096 | PV172610 |
|  | XG06926-15-2 | PV089196 | PV123097 | PV172611 |
| *C. homothallica* | CBS 168.53 ^T^ | JN205863 | JN206605 | KJ156498 |
|  | IFO 6736 | AF254941 | NA | NA |
| *C. inaequalis* | CGMCC3.28887 ^T^ | PV239670 | PV235914 | PV254883 |
|  | HZ156-2 | PV239671 | PV235915 | PV254884 |
| *C. intermedia* | CBS 347.69 | JN205892 | JN206606 | NA |
|  | IMI 200623 ^T^ | AF254939 | NA | NA |
| *C. irregularis* | CGMCC 3.16113 ^T^ | OL678145 | PQ399918 | NA |
|  | XY07657 | OL678146 | NA | NA |
| *C. monosporangiola* | CGMCC3.28888 ^T^ | PV239678 | PV235922 | PV785980 |
|  | HZ196-2 | PV239679 | PV235923 | PV785981 |
| *C. multiverticillata* | CBS 989.96 ^T^ | JN205897 | HM849693 | KJ156474 |
|  | Cu-137 ^T^ | AF254933 | NA | NA |
|  | NRRL 3009 | AF254943 | NA | NA |
| *C. nodosa* | Cu-34 ^T^ | AF346407 | NA | NA |
| *C. phaeospora* | CBS 692.68 ^T^ | JN205864 | HM849697 | NA |
| *C. polymorpha* | CBS 779.68 | JN205874 | JN206599 | NA |
| *C. regularis* | CGMCC 3.16114 ^T^ | OL678148 | PQ399919 | NA |
|  | XY07512 | OL678150 | NA | NA |
| *C. rhizoidea* | CGMCC 3.28654 | PV089205 | PV123106 | PV222157 |
|  | XG09702-9-2 | PV089206 | PV123107 | PV222158 |
| *C. saisamornae* | SDBR-CMUPFCM-6 | MW709394 | MW699571 | MW715866 |
|  | SDBR-CMU291 | MG571234 | MW699591 | MW715865 |
| *C. septata* | Cu-230 ^T^ | AF346408 | NA | NA |
| *C. simplex* | CGMCC 3.28653 | PV089201 | PV123102 | PV200767 |
|  | XG09611-12-2 | PV089202 | PV123103 | PV200768 |
| *C. subclavata* | CGMCC 3.16115 ^T^ | OL678152 | NA | NA |
|  | XY07766 | OL678153 | NA | NA |
| *C. tuberculata* | CGMCC3.28889 ^T^ | PV235918 | PV239674 | PV254887 |
|  | HZ162-2 | PV235919 | PV239675 | PV254888 |
| *C. varians* | CGMCC 3.16116 ^T^ | OL678154 | PQ399920 | NA |
|  | XY06999 | OL678155 | NA | NA |
| *C. vesiculosa* | CBS 989.96 ^T^ | JN205897 | HM849693 | KJ156474 |
|  | NRRL 3009 | AF254943 | NA | NA |
| *C. verrucosa* | CGMCC 3.16260 | ON262555 | ON261192 | NA |
|  | XY09506 | ON262556 | ON261193 | NA |
| *C. verticillata* | CBS595.68 ^T^ | AF254937 | NA | NA |
| *C. yunnanensis* | CGMCC 3.28655* | PV089207 | PV123108 | PV222159 |
|  | XG10042-9-2 | PV089208 | PV123109 | PV222160 |
| *Backusella oblongispora* | CBS 569.70 T | JN206251 | JN206407 | NA |

**Notes**: New species discovered herein are shown in bold. The asterisk "T" indicates the ex-type or ex-holotype strains. The "NA" stands for "not available".
